# PRESENTING VISION IMPAIRMENT AND RESIDENTIAL CARE USE AMONG OLDER ADULTS

**DOI:** 10.1093/geroni/igae098.4051

**Published:** 2024-12-31

**Authors:** Matthew Brault

**Affiliations:** NORC, Columbia, Maryland, United States

## Abstract

Nursing home and assisted-living facility use is common among older adults but is financially burdensome. Identifying risk factors for residential care use may focus attention on interventions that reduce or delay need for these services. Using data from the National Health and Aging Trends Study (NHATS) from 2021 (Round 11) and 2022 (Round 12), we measured the association of presenting visual acuity and contrast sensitivity on residential care use. Survey participants were assessed for presenting distance and near visual acuity and contrast sensitivity using a tablet-base examination tool. Residential care status, determined by the location of the Round 12 interview, was modeled as a function of vision impairment and relevant confounders using logistic regression. Of 2,727 participants, 2.1% were in residential care in 2022. Presenting near (19%) and distance acuity impairment (9%) were not associated with residential care, with adjusted odds ratios (aOR) of 1.48 (95% CI: 0.68 – 3.22) and 0.55 (0.17 – 1.81) respectively. Presenting contrast sensitivity impairment (24%) was associated with higher odds of residential care (aOR: 2.16 [95% CI 1.20 – 3.88]). This association was mediated by covariates for gait speed and recurrent falls. Results suggest that contrast sensitivity impairment may represent a separate risk factor for residential care use, apart from physical frailty or cognitive decline. Interventions to address or prevent eye diseases associated with impaired contrast sensitivity could reduce or delay residential care placement, a possibility that requires further investigation.

